# Safe to save blood in ovarian cancer surgery – time to change transfusion habits

**DOI:** 10.2340/1651-226X.2024.40435

**Published:** 2024-09-25

**Authors:** Anna Norbeck, Jesper Bengtsson, Susanne Malander, Mihaela Asp, Päivi Kannisto

**Affiliations:** a Department of Obstetrics and Gynecology, Skåne University Hospital, Lund University, Lund, Sweden; b Department of Clinical Science, Skåne University Hospital, Lund University, Lund, Sweden; c Clinical Immunology and Transfusion Medicine, Laboratory Medicine, Office for Medical Services, Region Skåne, Lund, Sweden; d Department of Oncology, Skåne University Hospital, Lund University, Lund, Sweden

**Keywords:** Ovarian cancer, patient blood management, red blood cell transfusion, intravenous iron, hemoglobin level

## Abstract

**Background:**

Patients with advanced ovarian cancer (AOC) undergoing surgery are often subjected to red blood cell (RBC) transfusions. Both anemia and RBC transfusion are associated with increased morbidity. The aim was to evaluate patient recovery after the implementation of patient blood management (PBM) strategies.

**Methods:**

This retrospective cohort study included 354 patients with AOC undergoing surgery at Skane University Hospital Lund, Sweden, between January 2016 and December 2021. The gradual implementation of PBM strategies included restrictive RBC transfusion, tranexamic acid as standard medication before laparotomies and intravenous iron administered to patients with iron deficiency. Severe complications were defined as Clavien-Dindo (CD) grade ≥ 3a. Logistic and linear regression analyses were used to evaluate the differences between three consecutive periods.

**Results:**

After the implementation of new strategies, 52% of the patients had at least one transfusion compared to 83% at baseline (*p* < 0.001). There was no difference in the rate of severe complications (CD ≥ 3a) between the groups, adjusted odds ratio 0.55 (95% CI 0.26–1.17). The mean difference in hemoglobin before chemotherapy was -1.32 g/L (95% CI -3.04 to -0.22) when adjusted for blood loss and days from surgery to chemotherapy. The length of stay (LOS) decreased from 8.5 days to 7.5 days (*p* 0.002).

**Interpretation:**

The number of patients transfused were reduced by 31%. Despite a slight increase in anemia rate, severe complications (CD ≥ 3a) remained stable. The LOS was reduced, and chemotherapy was given without delay, indicating that PBM is feasible and without causing major severe effects on short-term recovery.

## Introduction

Ovarian cancer is the eighth most common cancer in women worldwide and the most lethal gynecological malignancy [1]. Most ovarian cancer patients are diagnosed at an advanced stage and have a poor prognosis [2]. Complete cytoreduction of the tumor is highly important in advanced ovarian cancer (AOC) [3]. Surgery is therefore often extensive, enhancing intraoperative blood loss and consequently resulting in postoperative anemia [4, 5]. Perioperative red blood cell (RBC) transfusions are common in patients with AOC, with a transfusion rate of 40–77% [6, 7].

RBC transfusions have an immunosuppressive effect, and perioperative transfusions have been shown to be associated with an increased risk of cancer recurrence, morbidity and mortality [8–10]. Studies have shown reduced overall survival in patients with AOC receiving RBC transfusions compared with patients not receiving transfusions [9, 10]. In patients with AOC, perioperative transfusion is also associated with a prolonged length of stay (LOS) and increased morbidity but does not decrease overall survival or impact quality of life [6, 11]. Patient blood management (PBM) is a patient-centered approach for improving patient outcomes by preserving and managing patients’ own blood. PBM strategies have been implemented in many surgical settings and include detecting and treating preoperative anemia, minimizing blood loss and optimizing the patient’s physiological reserve [12].

Administration of tranexamic acid (TXA) reduces perioperative blood loss and the transfusion rate in patients undergoing elective abdominal and pelvic cancer surgery [13]. This is also true for patients with AOC undergoing cytoreductive surgery, and TXA does not increase the risk for postoperative complications. To optimize hemostasis, TXA is safe for patients and can be administered immediately before surgery [4, 5]. For these two reasons, the European Society of Gynecological Oncology guidelines recommend TXA peri-operatively and the correction of anemia [14].

Anemia is defined as hemoglobin (Hb) < 120 g/L for nonpregnant women and Hb < 130 g/L for men according to the World Health Organization [15]. To reduce RBC transfusion, it is recommended to treat anemia before surgery if Hb < 130 g/L, especially if the patient is undergoing major surgery and has an expected blood loss >500 mL [16].

Preoperative anemia is common among patients with advanced gynecological cancer, and a recent study showed that 42% of patients with AOC were anemic before surgery [17]. Iron deficiency anemia (IDA) is the most common cause of preoperative anemia in patients undergoing gynecological surgery [18]. Iron deficiency can be absolute with depleted iron reserves or functional with normal iron stores, but caused by cancer-induced inflammation and iron sequestration [19]. Patients receiving neoadjuvant chemotherapy have a greater rate of anemia prior to surgery and also a greater risk of preoperative transfusions [20]. In addition, preoperative anemia is associated with an increased risk of morbidity, such as increased risk for thromboembolic and infectious complications. Patients with anemia are also more likely to receive RBC transfusions [17]. Perioperative RBC transfusion does not seem to reduce the increased risk of morbidity [21].

Administration of intravenous iron perioperatively has been shown to reduce the need for RBC transfusion among patients undergoing major abdominal surgery [22]. A consensus statement by Shander et al. recommends screening for preoperative anemia and treating both pre- and postoperative IDA with intravenous iron [23]. Enhanced recovery after surgery society also recommends intravenous iron for treating anemia preoperatively in patients with gynecological cancer [24].

To our knowledge, there are no studies on the implementation of PBM strategies in patients with AOC undergoing surgery.

The primary aim of this study was to evaluate if RBC transfusions could be reduced after the gradual implementation of new PBM clinical routines and if it would affect the complication rate for the postoperative recovery of AOC patients. The secondary aim was to evaluate whether the Hb level before chemotherapy could be maintained when RBC transfusions were reduced.

## Materials and methods

This was a retrospective cohort study that included all patients with AOC, International Federation of Gynecology and Obstetrics (FIGO) stage III and IV, who underwent surgery, between January 1, 2016, and December 31, 2021, at the Department of Gynecology at Skane University Hospital Lund Sweden, a regional tertiary center for all gynecological malignancies. Patients with epithelial AOC, including fallopian tube and primary peritoneal cancer, who underwent surgery, either primary or interval debulking, were included. Patients who did not meet the criteria for having AOC and who had undergone debulking surgery were excluded. Thus, patients who underwent open-close surgery were excluded.

Inclusion criteria:

Advanced ovarian cancer

Primary or interval debulking surgery

Exclusion criteria:

Ovarian cancer FIGO stage I and II

Diagnoses other than ovarian cancer

Open-close cases

Patient data were collected from the electronic medical records of Region Skane, Melior (Melior.220-9.3.0.400–20210909.3, Cerner Corporation, Kansas City, USA) and Orbit (EVRY Healthcare System AB, Kristianstad, Sweden).

The data retrieved as continuous variables were age, body mass index (BMI), estimated blood loss, operating time, Hb level before surgery and before the start of chemotherapy, peri- and postoperative RBC transfusions, LOS and days from surgery to the start of chemotherapy. The categorical variables included histological diagnosis, FIGO stage, primary or interval debulking surgery (IDS), residual tumor, perioperative administration of TXA, administration of intravenous iron (ferric derisomaltose), surgical complexity score according to Aletti, the American Society of Anesthesiologists physical status classification system [25], the Eastern Cooperative Oncology Group (ECOG) performance status and postoperative complications at 30 days. Complications were classified according to the Clavien-Dindo (CD) classification [26]. If the patient had more than one complication, the most severe complication was registered. For the statistical analyses, the complications were grouped into two groups: no or minor complications (CD 0–2) and severe complications (CD 3–5). The surgical complexity score according to Aletti divided surgical procedures by complexity into three groups: low (3 or less), intermediate (4–7) or high (8 or more). Anemia was defined as a Hb level <120 g/L.

At the Department of Gynecology Skane University Hospital Lund, the following procedures were used to implement PBM clinical routines for patients with gynecological malignancies undergoing laparotomies: intravenous TXA 1,000 mg could be administered by a gynecologist’s order from 2016. It was introduced as a standard medication administered before major laparotomies in 2018. Screening for IDA before major surgery and the administration of intravenous iron to anemic patients with iron deficiency were implemented at the end of 2019. Patients with Hb <130 g/L and expected blood loss >500 mL with iron deficiency were also administered intravenous iron. Iron deficiency was defined as a TSAT < 0.20 or ferritin < 30mg/L. According to weight, patients were administered either a single dose of 1,000 mg or 1,500 mg of intravenous ferric derisomaltose (Monofer, Pharmacosmos, Denmark). During this period, guidelines for more restrictive RBC transfusions were gradually implemented, recommending a threshold for RBCs of Hb <70 g/L and for patients with comorbidities <80 g/L. RBCs and intravenous iron were administered by intravenous infusion before or after surgery according to the gynecologist’s decision to improve recovery. Intravenous iron was administered from 3 weeks to 1 day before surgery and during the postoperative period while the patient was in the hospital. RBC transfusions occurred either perioperatively or postoperatively while the patient was still in the hospital. Administration of intraoperative RBC transfusion was decided together by the surgeon and anesthesiologist. During this change of recommendation, RBC transfusion and intravenous iron administration were chosen by the senior consultant responsible for the patient.

### Statistical methods

For the statistical calculations, the study population was divided into three consecutive periods: year 2016–2017 (reference group), 2018–2019 and 2020–2021. For descriptive statistics, the chi-square test was used to compare categorical variables. One-way ANOVA test was used to compare means for continuous variables. As a nonparametric test, the Kruskal–Wallis test was used to compare median values among the three groups. Logistic regression was used to compare the CD grade among the three groups, unadjusted and adjusted for age, ECOG score, Aletti score and operating time. Linear regression was used to compare Hb levels before chemotherapy among the three groups, unadjusted and adjusted for estimated blood loss and number of days between surgery and chemotherapy. The data were analyzed using the statistical software IBM SPSS Statistics (Version 28.0. Armonk, NY: IBM Corp Data).

## Results

**Figure 1:**
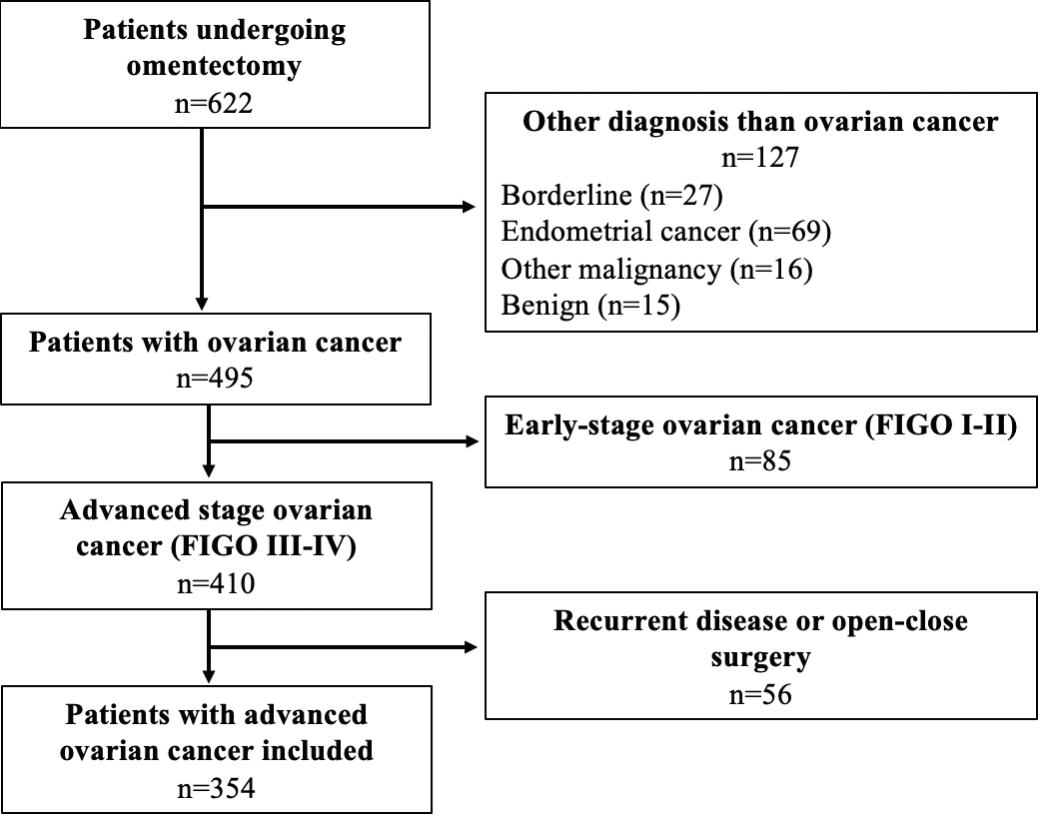
Flow chart of the study population.

**Figure 2:**
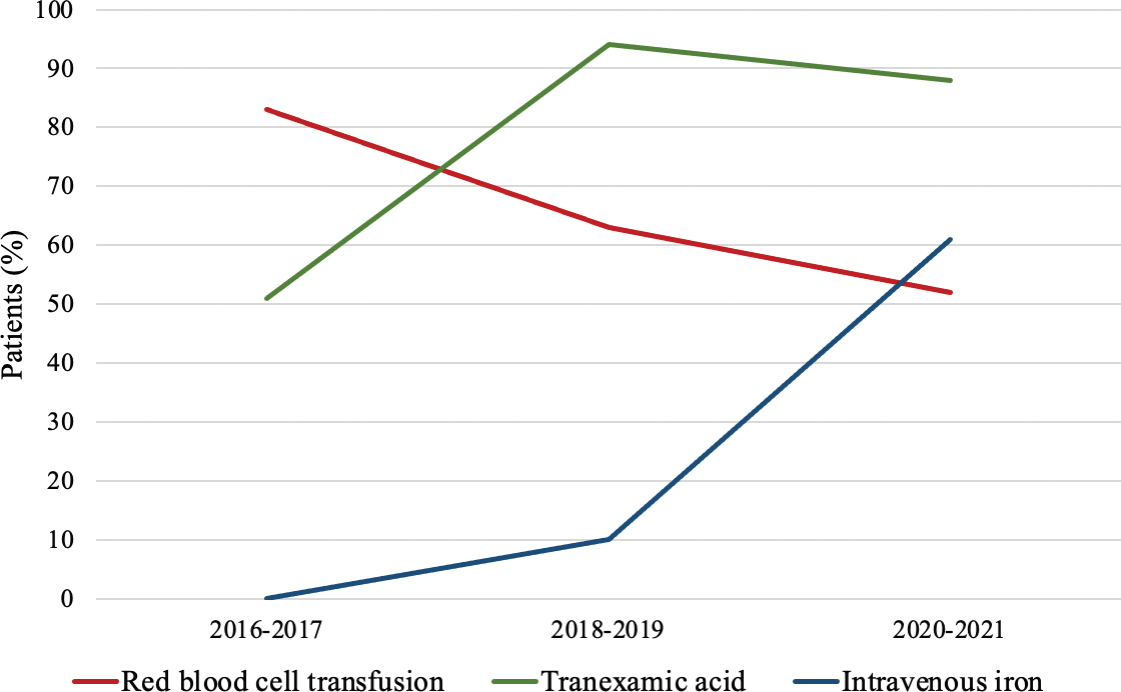
Gradual implementation of patient blood management clinical routines, including restrictive red blood cell transfusion, administration of tranexamic acid and administration of intravenous iron.

A total of 622 patients were identified, and 268 patients who did not meet the inclusion criteria were excluded. Thus, a total of 354 patients were included in the study, as shown in Figure 1. The patient characteristics are presented in Table 1. No differences were found in patient characteristics among the three groups, with a median age of 68–69 years and a preoperative Hb level between 120 and 123 g/L. The gradual implementation of new PBM clinical guidelines, including the administration of TXA and intravenous iron and the percentage of patients receiving RBC transfusions, is shown in Figure 2.

**Table 1:** Patient characteristics.

Variables	2016–2017 *n* = 114				2018–2019 *n* = 104				2020–2021 *n* = 136				*P*
	Median	IQR	*n*	%	Median	IQR	*n*	%	Median	IQR	*n*	%	
**Age (years) median IQR**	69	61–74			69	58–75			68	58–74			0.581
**FIGO**													0.551
III			91	80			78	75			101	74	
IV			23	20			26	25			35	26	
**Histology**													0.80
High-grade serous			102	90			86	83			123	90	
Low-grade serous			4	3			8	7			2	2	
Mucinous			3	3			2	2			5	4	
Endometroid			4	3			1	1			3	2	
Clear-cell			0	0			2	2			0	0	
Other			1	1			5	5			3	2	
**Mode of surgery**													0.019
Primary debulking surgery			94	83			69	66			105	77	
Interval debulking surgery			20	17			35	34			31	23	
**BMI (kg/m^2^) median IQR**	25	22–28			24	21–28			25	23–29			0.28
**ECOG**													0.111
0–1			113	99			100	96			128	94	
2–3			1	1			4	4			8	6	
**ASA**													0.566
1			23	20			24	23			24	23	
2			58	51			60	58			60	58	
3			32	28			20	19			36	26	
4			1	1			0	1			1	1	
**Preoperative Hb level (g/L) mean**	123.1 (95% CI 120.6–125.5)				120 (95% CI 117.2–122.9)				123 (95% CI 120.5–125.5)				0.201
**Preoperative anemia**													0.262
Hb <120 g/L			45	40			51	49			54	40	
Hb ≥ 120 g/L			69	60			53	51			82	60	
**Intravenous iron**													<0.001
Yes			0	0			10	10			83	61	
No			114	100			94	90			53	39	

FIGO: International Federation of Gynecology and Obstetrics; BMI: Body mass index; ECOG: The Eastern Cooperative Oncology Group performance status; ASA: The American Society of Anesthesiologists; Hb: Hemoglobin; IQR: Interquartile range.

**Figure 3:**
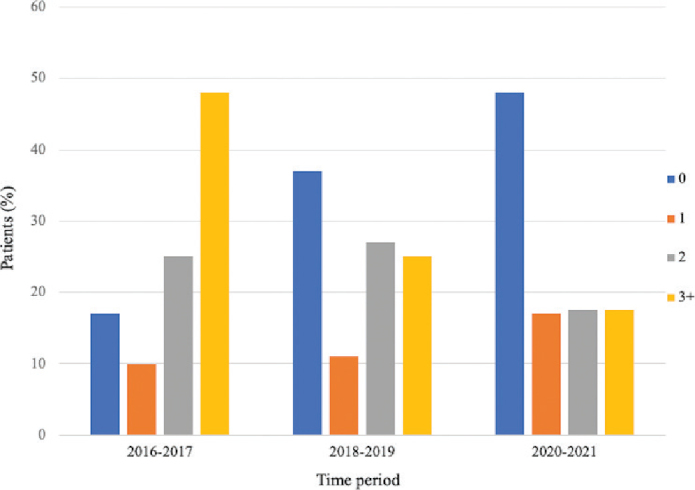
Percentage of patients transfused with units of red blood cells in the three consecutive periods.

After the implementation of PBM clinical routines, the percentage of transfused patients decreased from 83 to 52%, *p* < 0.001, as shown in Table 2. There was a significant decrease in the number of units per patient between the reference group and the 2020–2021 group (*p* < 0.001), as shown in Figure 3. The median length of hospital stay decreased from 8 to 7 days between the reference group and the 2018–2019 group (*p* < 0.001). There was no significant difference in the LOS between the 2018–2019 group and the 2020–2021 group. The operating time was reduced from a mean of 348 minutes to 214 minutes between the reference group and the 2020–2021 group. The estimated blood loss was also significantly reduced during this time period, as shown in Table 2.

**Table 2:** Patient characteristics surgery.

Variables	2016–2017 (*n* = 114)				2018–2019 (*n* = 104)				2020–2021 (*n* = 136)				*P*
	*n*	%	Median	IQR	*n*	%	Median	IQR	*n*	%	Median	IQR	
**Tranexamic acid**													<0.001
Yes	58	51			98	94			120	88			
No	56	49			6	6			16	12			
**Aletti score**													0.167
Low (3 or fewer)	42	37			40	39			41	30			
Intermediate (4–7)	47	41			52	50			66	49			
High (8 or more)	25	22			12	12			29	21			
**Residual tumor**													0.701
Radical	73	64			68	65			84	62			
<1 cm	35	22			21	20			37	27			
≥1 cm	16	14			15	14			15	11			
**Operating time (min) median IQR**			348	261–409			230	166–297			214	156–296	<0.001
**Blood loss (mL) median IQR**			600	400–1,100			400	200–800			500	300–800	<0.001
**RBC transfusion**													<0.001
Yes	95	83			65	63			71	52			
No	19	17			39	37			65	48			
**Units RBC transfused**	326				163				155				
**RBC transfusion n**													<0.001
0	19	17			39	37			65	48			
1	11	10			11	11			23	17			
2	29	25			28	27			24	17.5			
≥3	55	48			26	25			24	17.5			
**Perioperative RBC transfusion**													<0.001
Yes	70	61			43	41			46	34			
No	44	39			61	59			90	66			
**Clavien-Dindo classification**													0.050
0	1	1			7	7			14	10			
I	18	16			21	20			34	25			
II	67	59			63	60			71	52			
IIIa	15	13			6	6			11	8			
IIIb	6	5			3	3			4	3			
Iva	5	4			2	2			1	1			
IVb	1	1			0	0			0	0			
V	1	1			2	2			1	1			
**Severe complications**	28	25			13	12			17	12			0.17
**LOS (days) median IQR**			8.5	7–13			7.0	6–10			7.5	6–11	0.004
**Surgery to chemotherapy (days) median IQR**			33	27–39			28	22–36			24.5	21–31	<0.001
**Hb level before chemotherapy (g/L) mean**	118.9 (95% CI 117–120.8)				119 (95% CI 117–121.2)				115.2 (95% CI 113.4–117)				0.005
**Hb before chemotherapy PDS**	118.8 (95% CI 16.8–120.8)				120 (95% CI 117.3–122.7)				115.6 (95% CI 113.5–117.7)				0.017
**Hb before chemotherapy IDS**	119 (95% CI 113.3–124.7)				117.4 (95% CI 113.8–121)				113.8 (95% CI 110.3–117.4)				0.192

Aletti score surgical complexity score according to Aletti. IQR: Interquartile range; RBC: Red blood cell; LOS: Length of stay; Hb: Hemoglobin; PDS: Primary debulking surgery; IDS: Interval debulking surgery.

As shown in Table 3, in an unadjusted logistic regression model, the odds for CD ≥ 3 decreased during the time period of the study, with odds ratio (OR) 0.44 (95% CI 0.21–0.90) when comparing the 2018–2019 group with the reference group and OR 0.55 (95% CI 0.23–0.85) when comparing the 2020–2021 group with the reference group. In the adjusted model, it was no longer significant.

**Table 3:** Logistic regression analysis with unadjusted and adjusted odds ratios of Clavien-Dindo ≥3a comparing the 2018–2019 and 2020–2021 groups with the 2016–2017 reference group.

Year group	Unadjusted OR (95% CI)	*P*	Adjusted OR (95% CI)*	*P*
2016–2017	Reference	Reference	Reference	Reference
2018–2019	0.44 (0.21–0.90)	0.019	0.61 (0.28–1.32)	0.246
2020–2021	0.55 (0.23–0.85)	0.019	0.55 (0.26–1.17)	0.246

* Covariates: Age, The Eastern Cooperative Oncology Group (ECOG) performance status, surgical complexity score according to Aletti and operating time. OR: Odds ratio.

The mean Hb level before chemotherapy was 118.9 g/L (95% CI 117.0–120.8) in the reference group, 119.0 (95% CI 117.0–121.2) in the 2018–2019 group and 115.2 g/L (95% CI 113.4–117.0) in the 2020–2021 group (*p* 0.005). According to the adjusted linear regression model, there was a significant decrease in the Hb value from the reference group to 2020–2021, as shown in Table 4.

**Table 4:** Linear regression model for the difference in the hemoglobin level before chemotherapy among the three groups, both unadjusted and adjusted.

Year group	Unadjusted			Adjusted*		

	Coefficient	95% CI	*P*	Coefficient	95% CI	*P*
2016–2017	Reference			Reference		
2018–2019	0.18	-2.66 to 3.03	0.90	-0.10	-3.06 to 2.86	0.95
2020–2021	-1.83	-3.15 to -0.52	0.007	-1.32	-3.04 to -0.22	0.024
2018–2019	Reference			Reference		
2020–2021	-3.75	-6.64 to -1.06	0.007	-2.61	-5.43 to 0.22	0.07

* Adjusted for days from surgery to the start of chemotherapy and estimated blood loss.

## Discussion

Almost 50% of the patients were diagnosed with anemia before surgical intervention, which is comparable to the results reported by Foley et al. Patients with AOC have an increased risk of anemia at diagnosis compared with patients undergoing surgery for other gynecological malignancies and with those undergoing benign gynecological abdominal surgery [17].

After the implementation of PBM clinical routines, including TXA administration, restrictive strategies for RBC transfusion and intravenous iron administration to patients with IDA, the percentage of patients who did not receive RBC transfusion increased from 17 to 48%. The largest change was observed when TXA was introduced routinely, and the operating time decreased from 343 to 230 minutes. The Lymphadenectomy in Ovarian Neoplasm (LION) study randomized patients who achieved macroscopic radicality between systematic lymphadenectomy and the control. No survival benefits but heavy comorbidities were found when systematic lymphadenectomy was performed, indicating that this procedure could be omitted [27]. As a consequence of these results, the operating time in our study was shorter during the two later periods. RBC transfusions continued to decrease over the study period, and no negative effects, such as an increased number of complications or longer hospital stays, were observed. These transfusion rates are in line with what has been previously reported [6, 7]. This study included patients with AOC only, which might explain why the transfusion rate is higher both before and after the PBM strategies were implemented, compared with studies that also included patients with early-stage ovarian cancer [28]. Others have shown that patients receiving RBC transfusions often have a longer operating time and greater blood loss and are older than nontransfused patients [29]. However, in this study, no differences in median age between our three consecutive periods were found. Patients receiving perioperative RBC transfusions decreased from 61 to 34%, which could be a result of both shorter operating time and more restrictive strategies for RBC transfusions.

As shown in Table 2, the number of units transfused per patient also decreased. The greatest reduction (48 to 17%) was observed in the group of patients who received three or more units of RBCs. Transfusions with only one unit of RBC increased from 10 to 17%. A recent systematic review showed that perioperative RBC transfusion in patients undergoing elective major abdominal surgery was associated with an increased risk of postoperative mortality and morbidity, such as infectious complications [29]. In our study, severe complications seemed to decrease when RBC transfusions were reduced. However, the difference was not statistically significant when adjusted for operating time. Severe complications (CD ≥ 3a) were observed in 12–25% of patients, which is comparable to what has previously been reported [30]. During the study period, a sharp reduction in the number of patients transfused with more than one unit was observed. This indicates that physicians, as a clinical routine, likely accepted one unit strategy at a time, which is an important component of PBM implementation. After receiving only one allogenic transfusion, the patient is exposed to fewer foreign antigens and is likely to have a lower risk for adverse reactions. Despite the stable rate of severe complications, a decrease in the recovery time to chemotherapy of 8 days occurred simultaneously with the decrease in the number of RBC transfusions. This is important because severe complications are associated with delay in chemotherapy, which in turn affects patient survival [30]. The slightly lower Hb level at the start of chemotherapy did not seem to have any major clinical effect, probably because intravenous iron therapy was introduced and might have supported patient recovery. The effect of transfusion on complications and mortality is still unclear. A study by Hunsicker et al. [6] did not show an increased risk of cancer recurrence or mortality after transfusion [7]. Similar results were shown by Prescott et al., who reported that surgical site infections were more common in patients who were transfused.

The Hb level before chemotherapy decreased by 1.32 g/L, which is a small difference and is likely not clinically significant. According to the adjusted linear regression model, as shown in Tables 3 and 4, there was no significant difference between the reference group and the second group or between the second group and the last group in which all new clinical routines were implemented. Studies in colorectal cancer patients suggest that a single dose of intravenous iron may only partly elevate Hb, not normalize it, and may only temporarily fill the iron stores, which may explain why Hb is not normalized [31, 32]. Sustained iron repletion through monitoring of iron status and repeated intravenous iron treatment to achieve iron repletion has been shown to improve clinical and/or QoL outcomes in, for example, heart failure [33].

Similarly, during the last period, more than 60% of patients received iron intravenously, the majority before or few days after surgery. The median time from surgery to chemotherapy decreased from 33 days to 24.5 days toward the third period, which might have been too short for intravenous iron to increase the Hb level [23, 34]. A consensus statement by Shander et al. recommended screening for anemia at least 4 weeks before surgery for full effect, but screening shortly before surgery could also be valuable for the treatment of anemic patients with intravenous iron [23]. Treatment of anemia even 1 day before surgery has been shown to reduce RBC transfusions among patients with preoperative anemia [35]. However, no surgical studies have yet evaluated the impact of sustaining iron repletion throughout the entire patient treatment pathway. Almost all previous surgical studies have given a single dose of intravenous iron, as in this study. Recent publications on the dynamics of Hb and iron parameters after intravenous iron treatment in patients with colorectal cancer with IDA suggest that repeat treatment may be needed, as a substantial proportion of the patients relapse to IDA 4–8 weeks after treatment [31, 32]. This may also be relevant for patients with gynecological cancer, not least if they undergo repeat surgery and chemotherapy.

Patients who underwent IDS were more likely to be anemic both at the time of diagnosis and before surgery than patients who underwent primary debulking surgery (PDS) in a study by O’Shea et al. [20]. As shown in Table 2, there was a tendency toward lower Hb before chemotherapy in patients who underwent IDS when the number of perioperative transfusions decreased, although this difference was not statistically significant.

The median LOS decreased from 8.5 days to 7.5 days with reduced RBC transfusions. However, during the 6-year study period, the operating time decreased significantly, the estimated blood loss decreased and there were fewer severe complications, which all could have impacted the shorter LOS. The fact that RBC transfusion has been shown to be associated with longer hospital stays in patients who underwent ovarian cancer surgery is in line with the present study [11].

By implementing PBM as a clinical routine, including administering TXA before surgery, estimated blood loss was significantly reduced. It has previously been shown that single-dose TXA administered before surgery reduces blood loss and the transfusion rate in patients with AOC [4]. Similar results were shown by Yang et al., who used a higher dose of TXA [5]. In our study, 1,000 mg of TXA was used for all patients regardless of weight.

### Strengths and limitations

The strength of this study is that all patients included, during a six-year time period, had a primary diagnosis of AOC and underwent either PDS or IDS. Another strength is that all surgeries were conducted by experienced gynecological oncologists at a regional tertiary center for gynecological malignancies. This retrospective study has several limitations. Hb values were missing for patients who did not receive chemotherapy after surgery, and Hb values were also missing for some patients who did receive postoperative chemotherapy, for a total of 28 patients. The retrospective nature of the study leaves us ignorant of whether the patient had any symptoms of anemia after surgery, and we did not investigate whether the patients had any adverse transfusion reactions. Since we have described a structural change over a longer period of time, the individual gynecologist’s management has varied in time and per patient, even though PBM clinical routines were implemented. Confounders such as different surgical techniques might have affected the results. This study was not designed to analyze functional outcomes or fatigue, which could be the subject of further studies.

## Conclusion

In conclusion, it is safe to reduce RBC transfusions peri- and postoperatively in patients with AOC undergoing PDS or IDS. Despite a slight increase in the rate of anemia after the implementation of PBM clinical routines, severe complications (CD ≥ 3a) remained stable during the study period. The LOS was reduced, and chemotherapy was given without delay, indicating that implementing PBM strategies is feasible and has no major severe effects on short-term recovery or postoperative chemotherapy. Both the implementation of new guidelines and the cessation of old habits are time-consuming but unavoidable. Further prospective studies evaluating the effect of PBM interventions in patients with AOC are needed. There is also a need to investigate the potential differences between PBM interventions in patients undergoing PDS and those undergoing IDS and the role of intravenous iron in impacting patient recovery.

## Author contributions

AN: Methodology, investigation, formal analysis, writing original draft and review and editing. JB: Investigation, writing – review and editing. SM: writing – review and editing. MA: Investigation, writing – review and editing. PK: Conceptualization, methodology, investigation, formal analysis, writing – review and editing. All authors revised and reviewed the manuscript critically and gave final approval of the submitted version.

## Data Availability

The data that support the findings of this study are available upon request from the corresponding author.
